# Protective Role of Heme Oxygenase-1 in *Listeria monocytogenes*-Induced Abortion

**DOI:** 10.1371/journal.pone.0025046

**Published:** 2011-09-16

**Authors:** Masato Tachibana, Masanori Hashino, Takashi Nishida, Takashi Shimizu, Masahisa Watarai

**Affiliations:** 1 The United Graduate School of Veterinary Science, Yamaguchi University, Yamaguchi, Japan; 2 Department of Veterinary Public Health, Faculty of Agriculture, Yamaguchi University, Yamaguchi, Japan; Charité-University Medicine Berlin, Germany

## Abstract

It is well-known fact that various pathogens, including bacteria, virus, and protozoa, induce abortion in humans and animals. However the mechanisms of infectious abortion are little known. In this study, we demonstrated that *Listeria monocytogenes* infection in trophoblast giant cells decreased heme oxygenase (HO)-1 and B-cell lymphoma-extra large (Bcl-XL) expression, and that their overexpression inhibited cell death induced by the infection. Furthermore, HO-1 and Bcl-XL expression levels were also decreased by *L. monocytogenes* in pregnant mice. Treatment with cobalt protoporphyrin, which is known to induce HO-1, inhibited infectious abortion. Taken together, our study indicates that *L. monocytogenes* infection decreases HO-1 and Bcl-XL expression and induces cell death in placenta, leading to infectious abortion.

## Introduction

Listeriosis is caused by gram-positive *Listeria monocytogenes*. In humans, this pathogen has the ability to cross the intestinal, placental, and blood-brain barriers, leading to gastroenteritis, maternofetal infections, and meningoencephalitis, respectively. A key feature of the virulence of *L. monocytogenes* is its ability to avoid the killing mechanisms of professional and non- professional phagocytic host cells [1]–[4]. *L. monocytogenes* infections in humans are caused mainly by injection of contaminated food, such as daily products, raw vegetables, fish, poultry, processed chicken, and beef [5].

*L. monocytogenes* induces cell death *in vitro* and *in vivo* in various cell types including hepatocytes [6], lymphocytes [7], and dendritic cells [8]. Cell death induced by *L. monocytogenes* is associated with listeriolysin O, a pore-forming toxin that allows bacteria to lyse the phagosomal membrane and escape into the cytosol.

In a previous study, we investigated abortion induced by brucella infections and demonstrated that it was associated with cell death of placental immune cells, the trophoblast giant (TG) cells. Furthermore, we found that heme oxygenase (HO)-1 expression inhibited infectious abortions *in vivo* and cell death *in vitro* [9]. HO-1 plays a key role in cytoprotection, anti-oxidation, and anti-inflammation. Most of the physiological functions of HO-1 are associated with its enzymatic activity in heme catabolism [10], [11]. In humans, HO-1 deficiency is associated with susceptibility to oxidative stress and an increased pro-inflammatory state, leading to severe endothelial damage [12]. Mice lacking HO-1 develop progressive inflammatory disease [13] and show enhanced lipopolysaccharide-induced toxemia [14]. Although the protective properties of HO-1 have been studied using various inflammatory models [15]–[20], the molecular mechanisms, timing, and mode of HO-1 function during disease remains largely unknown. HO-1 expression is known to be associated with B-cell lymphoma-extra large (Bcl-XL) expression [21]. Bcl-XL is one of the several anti-apoptotic proteins that are members of the Bcl-2 family [22].

*L. monocytogenes* infection causes abortion in pregnant mice [23]. However, the factors involved in abortion induced by *L. monocytogenes* infection in these animals remain unknown. In the present study, we investigated the roles of the anti-apoptotic factors, HO-1 and Bcl-XL, in abortion induced by *L. monocytogenes* infection. HO-1 and Bcl-XL expression was down-regulated by *L. monocytogenes* infection or interferon (IFN)-γ treatment, leading to infectious abortion. HO-1 and Bcl-XL overexpression suppressed this infectious abortion. These results suggest that HO-1 and Bcl-XL play a critical role in the control of infectious abortion induced by *L. monocytogenes*.

## Results

### *L. monocytogenes* infection decreased HO-1 and Bcl-XL expression in TG cells

**Figure 1:**
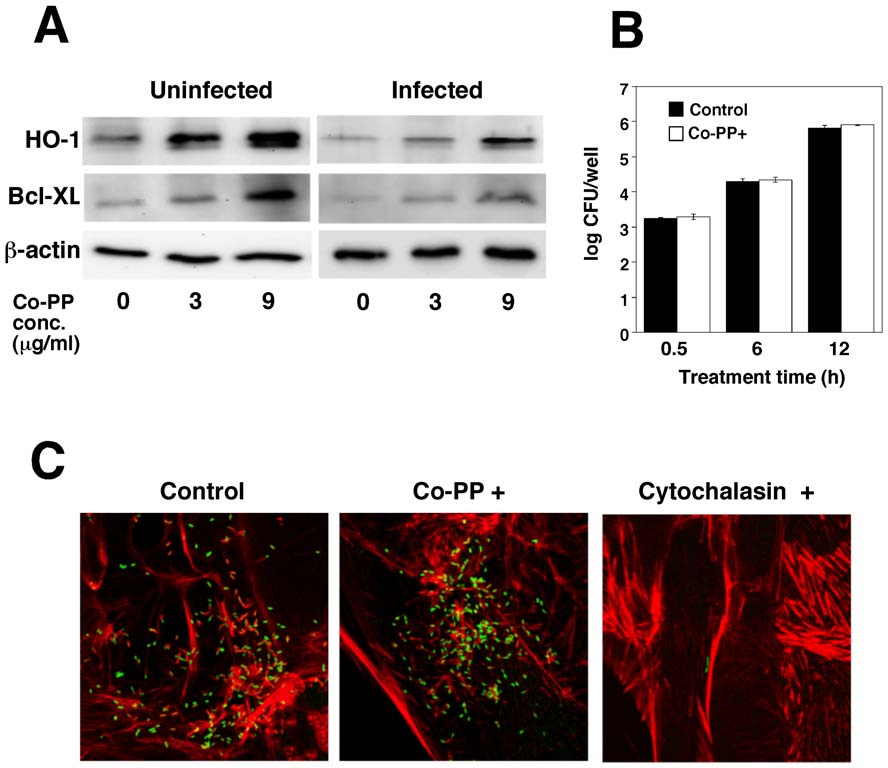
Decreased HO-1 and Bcl-XL expression in TG cells infected with *L. monocytogenes*. (A) TG cells were first treated with Co-PP and then infected with *L. monocytogenes*. The infected cells were cultured in 50 µg/ml of gentamicin. After 6 h, HO-1 and Bcl-XL expression was analyzed by immunoblotting. A representative immunoblot of three independent experiments is shown. (B) TG cells were treated with Co-PP and then infected with *L. monocytogenes*. The infected cells were cultured in 50 µg/ml of gentamicin. After 0.5, 2, and 6 h incubation, the infected cells were washed with PBS and lysed with cold distilled water. CFU were determined by serial dilution on BHI agar plates. (C) *L. monocytogenes* was deposited on TG cells by centrifugation at 150×g for 10 min at room temperature, incubated for 6 h, fixed, and stained. The figure shows FITC-labeled bacteria (green) and Alexa Fluor 594-labeled actin filaments (red) merged images. The left-hand panel shows untreated cells, the center panel Co-PP (9 µg/ml)-treated cells, and the right-hand panel, cytochalasin D-treated cells.

*L. monocytogenes* has been shown to infect the placenta and induce cell death in vitro and in vivo [24]–[26]. TG cells are placental immune cells existing in maternal-fetal interface and these cells are important for maintaining pregnancy [27]. In a previous study, we demonstrated that HO-1 plays a role in inhibiting cell death induced by *Brucella abortus* infection. To investigate the mechanisms through which *L. monocytogenes* induces cell death in placenta, we measured HO-1 expression in TG cells. HO-1 was expressed in TG cells, but its expression decreased on *L. monocytogenes* infection (Fig. 1A). Furthermore, HO-1 expression was enhanced by the HO-1 inducer cobalt protoporphyrin (Co-PP), in a concentration-dependent manner (Fig. 1A). No significant difference was observed in intracellular growth of bacteria between Co-PP-treated and non-treated TG cells (Fig. 1B, C). These results indicate that *L. monocytogenes* infection decreases HO-1 expression. To investigate the mechanism of HO-1, Bcl-XL expression was analyzed (Fig. 1A). Bcl-XL, an anti-apoptotic protein induced by HO-1, belongs to the Bcl-2 family [28], [29]. Bcl-XL expression was enhanced by the HO-1 inducer Co-PP and decreased by *L. monocytogenes* infection as well as HO-1. Furthermore, we showed that this reduction in expression was recovered by Co-PP.

**Figure 2:**
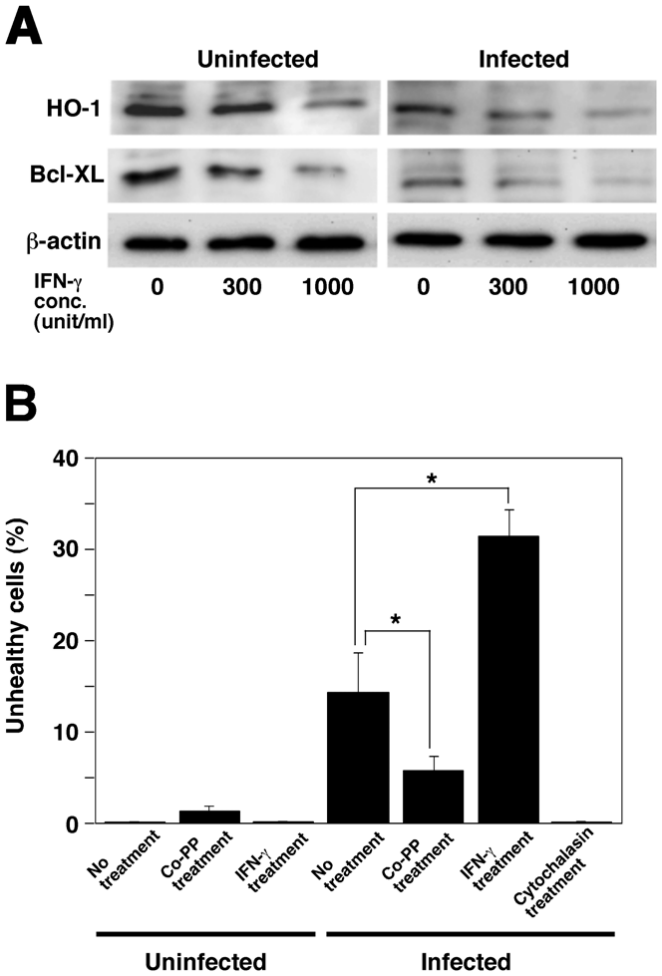
Induction of cell death by *L. monocytogenes* infection. (A) TG cells were treated with IFN-γ (0, 300, and 1,000 units/ml) for 24 h and infected with *L. monocytogenes* for 6 h. HO-1 and Bcl-XL expression in TG cells was analyzed by immunoblotting. A representative immunoblot of three independent experiments is shown. (B) Cell death was determined using the JC-1 Mitochondrial Membrane Potential Assay Kit. One hundred TG cells per coverslip were examined to determine the total number of live or dead cells. All values represent the average and the standard deviation of three identical experiments. Statistically significant differences compared with the control are indicated by asterisks (*, P<0.05).

Since an increase in IFN-γ due to *L. monocytogenes* infection was observed to promote abortion in pregnant mice [30], we investigated the effect of IFN-γ treatment on HO-1 and Bcl-XL expression in TG cells. HO-1 and Bcl-XL expression in TG cells decreased significantly in a concentration-dependent manner on treatment with IFN-γ, with the down-regulation being enhanced further by *L. monocytogenes* infection (Fig. 2A).

### HO-1 and Bcl-XL protect against cell death induced by *L. monocytogenes* infection

To examine whether HO-1 and Bcl-XL inhibited cell death, TG cells were infected with *L. monocytogenes* with or without Co-PP treatment and the rate of cell death was determined measuring mitochondrial membrane potential. Mitochondrial membrane potential has been used as an indicator of cell death. In this experimental system, cell death induced cells with low mitochondrial membrane potential were detected as unhealthy cells (Fig. 2B). Treatment with Co-PP inhibited cell death induced by *L. monocytogenes* infection in TG cells as compared with untreated TG cell. In contrast, cell death induced by *L. monocytogenes* infection in IFN-γ-treated TG cells was enhanced compared to that in untreated TG cells (Fig. 2B). Treatment with cytochalasin D, which is known to inhibit *L. monocytogenes* internalization, was found to inhibit the death of TG cells by *L. monocytogenes* infection compared with non-treated TG cells (Fig. 2B). These results indicate that internalization of *L. monocytogenes* decreases HO-1 and Bcl-XL expression leading to enhancement of cell death.

**Figure 3:**
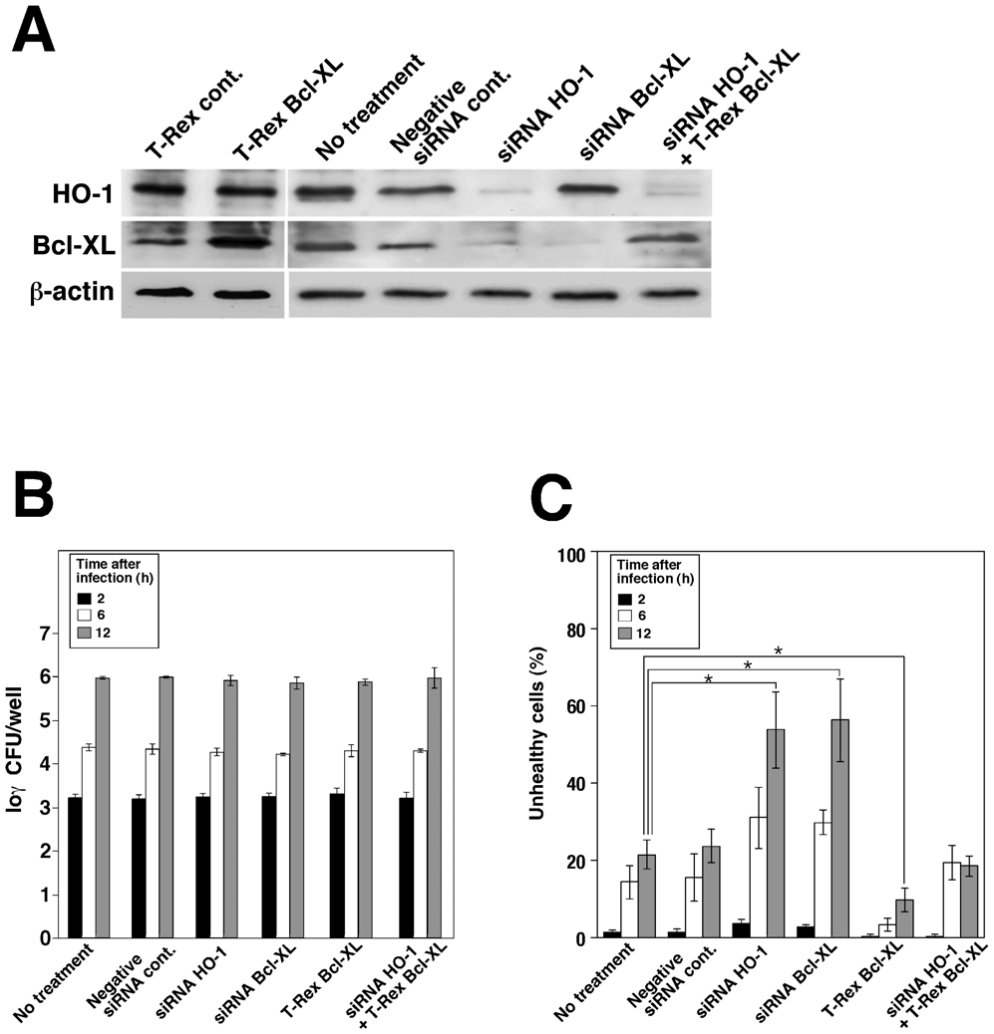
Prevention of cell death by HO-1 and Bcl-XL expression. (A) TG cells were treated for 48 h with either siRNA targeting HO-1, Bcl-XL, or control siRNA (QIAGEN AllStars Negative Control). Bcl-XL overexpression was achieved by transfecting the cells with pcDNA4/TO-Bcl-XL. HO-1 and Bcl-XL expression was monitored by immunoblotting. β-actin was used as an internal control. A representative immunoblot of three independent experiments is shown. (B) TG cells were infected with *L. monocytogenes*. The infected cells were cultured with media containing 50 µg/ml gentamicin for 2, 6, and 12 h. The cells were then washed with PBS and lysed with cold distilled water. CFU was determined by serial dilution on BHI agar plates. All values represent the average and the standard deviation of three identical experiments. (C) Cell death was determined using the JC-1 Mitochondrial Membrane Potential Assay Kit. One hundred TG cells per coverslip were examined to determine the total number of live or dead cells. All values represent the average and the standard deviation of three identical experiments. Statistically significant differences compared with the control are indicated by asterisks (*, P<0.05).

To confirm the effect of HO-1 and Bcl-XL on TG cell death following infection with *L. monocytogenes*, we reduced the amount of endogenous HO-1 and Bcl-XL by transfecting HO-1-specific small interfering RNA (siRNA) duplexes into TG cells. After 48 h of transfection with HO-1-specific siRNA, HO-1 and Bcl-XL expression levels were no longer detectable, but were not affected by transfection with β-actin or control siRNA (Fig. 3A). HO-1 or Bcl-XL knockdown did not induce cell death in TG cells (Fig. 3C). While *L. monocytogenes* infection resulted in a slight induction of cell death in TG cells, HO-1 or Bcl-XL knockdown enhanced cell death in infected TG cells (Fig. 3C). Bcl-XL overexpression in the T-Rex system inhibited cell death compared to cells not expressing the protein (Fig. 3C). There was no significant difference in bacterial growth between transfected and non-transfected TG cells (Fig. 3B). These results suggest that HO-1 and Bcl-XL play critical roles in the inhibition of cell death induced by *L. monocytogenes* infections.

### Abortion induced by *L. monocytogenes* infection is dependent on HO-1 and Bcl-XL expression in the placenta

**Figure 4:**
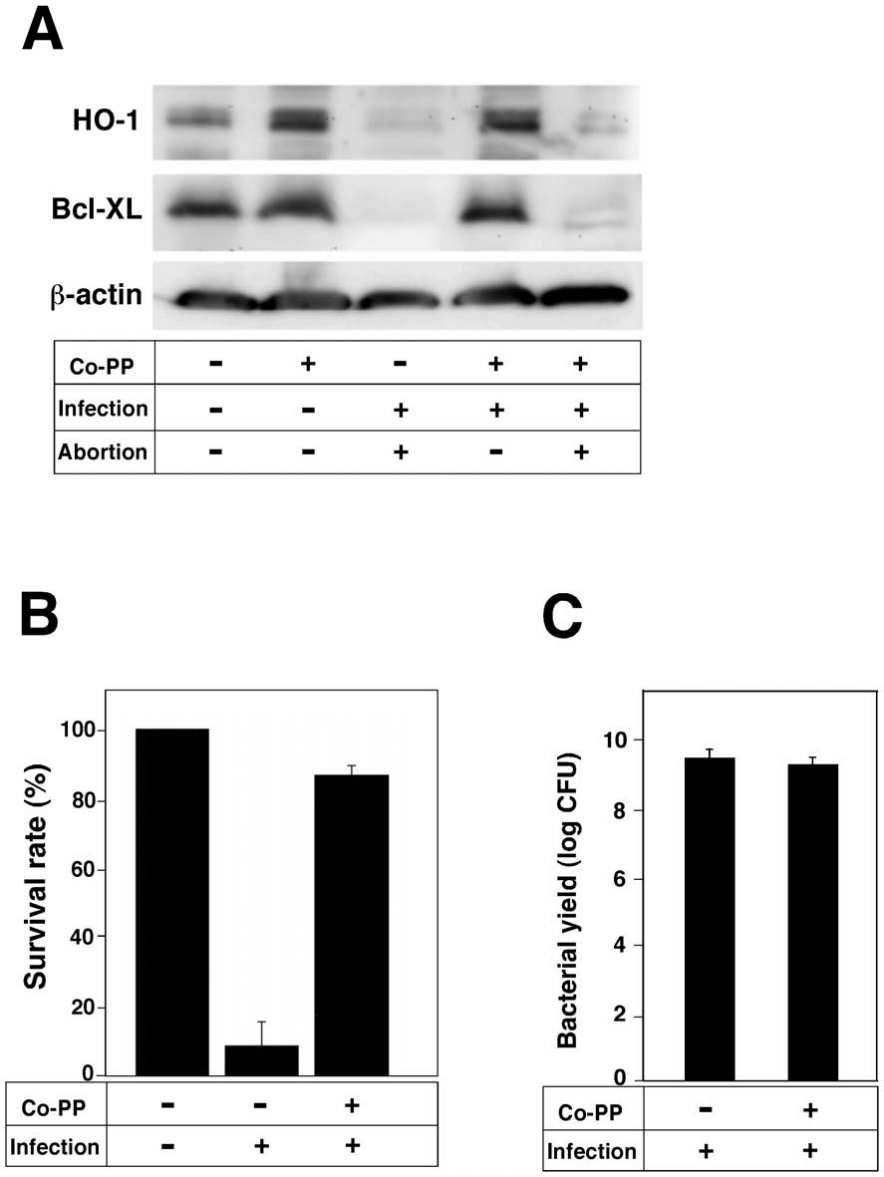
Prevention of infectious abortion by HO-1 and Bcl-XL expression. (A) Pregnant mice were infected with 10^5^ cells of *L. monocytogenes* in 0.1 ml of saline at day 13.5 of pregnancy with or without Co-PP treatment (5 mg/kg). At day 16.5, the placentas, fetuses, and livers were removed. HO-1 and Bcl-XL expression in the placenta was analyzed by immunoblotting. A representative immunoblot of three independent experiments is shown. (B) Survival rates were determined by the presence or absence of a heartbeat in the fetuses. (C) Livers were homogenized in saline and diluted with PBS. CFU was determined by plating the diluted samples on BHI agar plate.

Previous studies have reported the presence of HO-1 in the mammalian placenta and postulated that it has a protective role during pregnancy [31]–[33]. We assume that the inhibitory action of HO-1 and Bcl-XL on cell death leads to a successful pregnancy. To examine whether HO-1 and Bcl-XL actually block abortion induced by *L. monocytogenes* infection, we measured HO-1 and Bcl-XL expression levels in the placenta of *L. monocytogenes*-infected mice. Both HO-1 and Bcl-XL were expressed in the placenta of mice, with levels being decreased by *L. monocytogenes* infection (Fig. 4A). Moreover, injection of *L. monocytogenes* with Co-PP restored HO-1 and Bcl-XL expression levels (Fig. 4A). We next investigated the role of HO-1 and Bcl-XL expression on abortion induced by *L. monocytogenes*. Infection of *L. monocytogenes* induced abortion in pregnant mice (Fig. 4B). HO-1 and Bcl-XL expression induced by Co-PP injection blocked abortion in *L. monocytogenes*-infected mice (Fig. 4B). There was no significant difference in the growth of bacteria in livers (Fig. 4C) and placenta (data not shown) between Co-PP-treated and untreated mice. These results suggest that abortion induced by *L. monocytogenes* infection is dependent on HO-1 and Bcl-XL expression in the placenta.

## Discussion

Heme oxygenases (HOs) are heme catabolic enzymes. Heme is degraded to carbon monoxide, biliverdin, and ferrous ion. Biliverdin is converted to bilirubin, which is believed to be a potent anti-oxidant. Three isoforms of HOs have been identified. HO-1 is an inducible isoform produced in response to various types of stress, such as oxidative stress, heat stress, endotoxin stress, hypoxia, heavy metal stress, and cytokine stress [34]. Furthermore, HO-1 plays a role in cytoprotection, anti-oxidation, anti-inflammation, and graft acceptance [35]–[37]. HO-1 is also down-regulated at the fetal maternal interface during spontaneous abortion in both humans and mice [33], [38]–[40]. Up-regulation of HO-1 by Co-PP prevents abortion, while down-regulation by zinc protoporphyrin increases the chances of abortion [31]. It has been reported that during pregnancy, all placental cell types are positive for HOs and that different types of trophoblast cells are important sources of these enzymes [32], [38], [40], [41]. As we anticipated, HO-1 was associated with infectious abortion. It is well known that various pathogens, such as *Brucella* spp., *L monocytogenes*, *Leptospira* spp., *Buniyavirus*, and *Toxoplasma gondii*, cause infectious abortion. However, the mechanisms responsible for infectious abortion remain unclear. Previously, we reported that HO-1 was associated with abortion induced by *B. abortus* infection. *B. abortus* are gram-negative, intracellular, and zoonotic bacteria that cause down-regulation of HO-1 in the placenta leading to abortion. However, it remains unclear whether HO-1 is a common regulator for abortion induced by various pathogens. In this study, we used gram-positive, intracellular, and zoonotic *L. monocytogenes* to examine this possibility.

In order to investigate the detailed mechanisms of infectious abortion induced by *L. monocytogenes*, we studied TG cells *in vitro*. TG cells are immunocompetent cells present in the placenta [42]–[44] and play a critical role in implantation and pregnancy [27], [42]. HO-1 expression in TG cells was decreased by *L. monocytogenes* infection (Fig. 1A) and treatment with IFN-γ (Fig. 2A). Furthermore, it is well known that IFN-γ is induced by *L. monocytogenes* infection in mice [30] and there is evidence that Th1 cytokines, such as IFN-γ, inhibit HO-1 expression resulting in allograft rejection [37]. These results indicate that Th1 cytokines induced by *L. monocytogenes* infection control HO-1 expression.

Although HO-1 appears to play a critical role in the control of infectious abortion, the mechanisms of this control remain unclear. We focused on Bcl-XL since HO-1 enhances the expression of this anti-apoptotic protein [21]. We found that Bcl-XL expression was enhanced by a HO-1 inducer, Co-PP (Figs. 1A, 4A) and furthermore that Bcl-XL overexpression prevented cell death induced by *L. monocytogenes* infection (Fig. 3C). These results suggest that Bcl-XL is a key factor that protects placenta cells from injury induced by *L.* monocytogenes infection, thereγy resulting in successful pregnancy.

We also observed that HO-1 and Bcl-XL expression was down-regulated in the placenta of pregnant mice by *L. monocytogenes* infection (Fig. 4A), while it was up-regulated by Co-PP and inhibited infectious abortion (Fig. 4B). These results suggest that HO-1 and Bcl-XL have critical roles against infectious abortion induced by *L. monocytogenes*.

Although HO-1 and Bcl-XL play an important role to protect cells from cell death, it is still unknown how HO-1 and Bcl-XL inhibit cell death induced by *L. monocytogenes* infection. Caspase-9 is an apoptotic protein and its activation is inhibited by Bcl-XL [45]. In TG cells, however, *L. monocytogenes* infection failed to induce caspase-9 activation (data not shown). These results may indicate that *L. monocytogenes* induces cell death trough alternative pathways involved with Bcl-XL.

In humans, it was reported that *L. monocytogenes* infects extravillous trophoblasts (EVTs), and spreads across maternal-fetal barrier [46]. However, there is less information about molecular mechanisms by which *L. monocytogenes* passes maternal-fetus barrier. Since trophoblast cells such as EVTs in human or TG cells in mouse exists in maternal-fetal interface, down regulation of HO-1 and Bcl-XL leading to enhancement of cell death may be a key event for *L. monocytogenes* to spread across the barrier.

In conclusion, our results indicate that down-regulation of HO-1 induced by various pathogens may be a key event in infectious abortion. Antimicrobial drugs are usually used in the treatment of listeriosis. However, an increasing number of multidrug-resistant *L. monocytogenes* have been reported [47], [48]. It is noteworthy that the HO-1 inducer Co-PP suppressed abortion induced by *L. monocytogenes*. Therefore, HO-1 has potential as a putative therapeutic target in infectious abortion.

## Methods

### Bacterial strains

*L. monocytogenes* EGD was maintained as a frozen glycerol stock and cultured in brain heart infusion (BHI) broth (Becton Dickinson) or on BHI broth containing 1.5% agar.

### Cell culture

Mouse trophoblast stem (TS) cell line was gifted from Dr. Tanaka [44], [49]. TS cells were cultured in TS medium in the presence of fibroblast growth factor-4, heparin, and mouse embryonic fibroblast-conditioned medium as described previously [49]. The TS medium was prepared by adding 20% fetal bovine serum, 1 mM sodium pyruvate, 100 µM β-mercaptoethanol, and 2 mM L-glutamine to RPMI 1640. To induce differentiation to TG cells, the cells were cultured in TS medium alone for 3 days at 37°C in a CO_2_ incubator. The cells were then seeded in a 48-well (1–2×10^5^ per well) or a 12-well (4–8×10^5^ per well) tissue culture plate.

### Immunoblotting

The protein samples were separated on a 15% polyacrylamide gel and transferred to a polyvinylidene difluoride membrane, which was incubated for 16 h at 4°C with anti-HO-1 rabbit polyclonal antibody (Stressgen) or anti-Bcl-XL rabbit polyclonal antibody (Cell Signaling Technology) at a dilution of 1∶5000 or 1∶1000 in 5% skim milk. The membrane was washed three times in Tris-buffered saline with 0.02% Tween 20, incubated for 30 min with 0.01 µg/ml horseradish peroxidase-conjugated secondary antibody, and washed again. The immunoreactions were visualized using the enhanced chemiluminescence detection system (GE Healthcare Life Science). The β-actin antibody was purchased from Sigma.

### Efficiency of bacterial replication within cultured cells

*L. monocytogenes* strains were deposited onto TG cells at a multiplicity of infection (MOI) of 10 by centrifugation at 150×g for 10 min at room temperature. To measure the intracellular replication efficiency, the infected cells were incubated at 37°C for 30 min, washed once with TS medium, and then incubated in TS medium containing gentamicin (50 µg/ml) for 0.5, 2, 6, and 12 h. The cells were washed three times with phosphate-buffered saline (PBS) and lysed with cold distilled water. Colony forming unit (CFU) was determined by serial dilution on BHI agar plates. Cytochalasin D (Wako), recombinant IFN-γ (Cedarlane Laboratories) or Co-PP was added to the TS medium at the indicated concentrations 2, 16, and 24 h before infection.

### Immunofluorescence microscopy

Bacteria were deposited onto TG cells grown on coverslips by centrifugation at 150×g for 5 min at room temperature and were then incubated at 37°C for 30 min. The samples were washed twice with PBS and fixed with 4% paraformaldehyde in PBS for 30 min at room temperature, washed three times with PBS, and incubated successively three times for 5 min in blocking buffer (5% bovine serum albumin in PBS) at room temperature. The samples were permeabilized in 0.2% Triton X-100 and washed three times with PBS, followed by treatment with 5 µg/ml anti-*L. monocytogenes* polyclonal rabbit antibody (Viro Stat) diluted in blocking buffer to identify intracellular bacteria. After incubation for 1 h at 37°C, the samples were washed three times for 5 min with blocking buffer, stained with FITC-labeled goat anti-rabbit IgG (0.01 µg/ml, Chemicon) in blocking buffer, and incubated for 1 h at 37°C. Fluorescent images were obtained using a FluoView FV100 confocal laser scanning microscope (Olympus).

### Expression of recombinant protein

Total RNA was isolated from TG cells using the RNA Purification Kit (Qiagen), and the purified RNA samples were stored at −30°C until use. RNA was quantified by absorption at 260 nm using the SmartSpec 3000 spectrophotometer (Bio-Rad). RT-PCR was performed using Superscript II Kit (Invitrogen). The primers used for mouse Bcl-XL amplification were 5′- ATGTCTCAGAGCAACCGGG AG -3′ and 5′- TCACTTCCGACTGAAGAGTGA -3′. To express Bcl-XL in TG cells, amplified DNA encoding Bcl-XL from TG cells in RT-PCR was cloned into the pcDNA4/TO vector of the T-Rex System (Invitrogen). pcDNA4/TO-Bcl-XL was transfected into TG cells using the FuGENE 6 Transfection Reagent (Roche) at a final concentration of 1.2 µg/ml.

### siRNA experiment

siRNA duplexes used for silencing mouse HO-1 (target sequence: CAGCCACACAGCACTATGTAA) and Bcl-XL (target sequence: AAAGTGCAGTTCAGTAATAAA) and AllStars Negative Control siRNA were purchased from QIAGEN. TG cells were transfected transiently using the X-tremeGENE siRNA Transfection Reagent (Roche) with or without a final concentration of 10 nM for siRNAs.

### Determination of cell death

Cell death was determined using the JC-1 Mitochondrial Membrane Potential Assay Kit (Cayman Chemical) according to the manufacturer's instructions. Mitochondrial membrane potential, DJm, an important parameter of mitochondrial function, is used as an indicator of cell health. Healthy cells have a high mitochondrial DJm and red fluorescence, while apoptotic or unhealthy cells have a low DJm and green fluorescence [50].

### Mice

Six to 10-week-old BALB/c female mice were mated individually to 6- to 10-week-old BALB/c male mice. The parent mice were obtained from Kyudo Co., Ltd.. Vaginal plug was observed at day 0.5 of gestation. The normal gestational time for these mice is 19 days.

### Virulence in pregnant mice

Groups of five pregnant mice were infected intraperitoneally at 13.5 days of gestation with approximately 10^5^ cells of *L. monocytogenes* in 0.1 ml saline with or without Co-PP (5 mg/kg, Sigma). On day 16.5 of gestation, their livers were removed and homogenized in saline. The tissue homogenates were serially diluted with PBS and plated on BHI agar plates to estimate the number of CFU. Fetuses were classified as alive if there was a heartbeat and as dead if there was no heartbeat. The animal experiments were approved by the Animal Research Committee of Yamaguchi University (permit number: 141).

### Statistical analyses

Statistical analyses were performed using Student's t test. Statistically significant differences compared with the controls are indicated by asterisks (*, P<0.05). Data are expressed as the mean of triplicate samples from three identical experiments, and the error bars represent the standard deviations.
